# Myocardial lesion in patients with COVID-19: Not all is in the lung

**DOI:** 10.1016/j.jtumed.2021.01.006

**Published:** 2021-02-05

**Authors:** Ivan D. Lozada-Martinez, Daniela M. Torres-Llinás, Luis R. Moscote-Salazar

**Affiliations:** a Department of Medicine, Medical and Surgical Research Center, School of Medicine, University of Cartagena, Cartagena, Colombia; b Colombian Clinical Research Group in Neurocritical Care, School of Medicine, University of Cartagena, Cartagena, Colombia

To the Editor:

The COVID-19 pandemic has demonstrated that the severe acute respiratory syndrome coronavirus 2 (SARS-CoV-2) infectious agent has an overwhelmingly highly virulent capacity.^1^ Case studies with representative samples have found that the inflammatory process is not only located at the lung level. In most cases, the patient's death is due to complications resulting from other white organ failures such as the heart, endothelium, kidneys or brain.^2^ Since the Middle East respiratory syndrome (MERS) epidemic, it became evident that coronaviruses can induce myocarditis and heart failure in numerous patients. Considering that SARS-CoV-2 and MERS have similar pathogenicities,^3^ the prognoses of patients with basic cardiovascular diseases were expected to be reflected in the high lethality rates currently observed in this group. These rates result from functional and structural alterations of the cardiovascular system, in addition to the difficulty of establishing effective therapies.

Evidently, the degree of cardiac involvement is directly proportional to the severity of the condition. Indeed, higher levels of troponin I, blood pressure, and creatine phosphokinase myocardial band (CPK-MB) were found in patients who were admitted to intensive care units than in those who were not.^4^ However, myocardial injury markers prevail, even in those with mild symptoms.^4^ Moreover, in some cases, a presumptive COVID-19 diagnosis was established based on cardiovascular symptoms rather than pulmonary symptoms, such as heart palpitations and chest pain, in the absence of cough, fever, or other respiratory symptoms.^3^ As substantial cardiovascular abnormalities have been found in this type of patients, even in cases which do not present cardiovascular risk factors, they are becoming an independent morbidity-mortality predictor.^5^

Lippi et al.^6^ performed a systematic review and meta-analysis in which they evaluated troponin I levels in 374 patients with COVID-19 disease. In doing so, they found that severely ill patients had significantly higher troponin I levels than patients with the non-severe form of the disease (OR 25.6, 95% CI: 6.8–44.5).^6^ Nevertheless, mechanisms by which a troponin I elevation can occur have been described, without necessarily being secondary to an ischemic process of the myocardium, including severe hypoxia, sepsis, systemic inflammation, pulmonary thromboembolism, and stressful cardiomyopathy.^7^ However, many of these conditions can lead to ischemia and myocardial infarction. Conversely, there is doubt on whether the pathophysiology is linked to the presence of the virus in the myocardial tissue. Indeed, studies have found an inflammatory infiltrate consisting of mononuclear cells during the autopsy of patients who died from fulminating myocarditis and who expressed a high viral load. However, they did not confirm the presence of the virus.^8^ Thus, complex and large-scale studies must be carried out to evaluate the evidence in detail.

So far, two mechanisms by which myocardial lesion is induced secondary to COVID-19 have been described. The first is related to the dynamics of the angiotensin-converting enzyme - 2 (ACE2), which is expressed at the lung level as well as in the heart. Here, the internalisation of the virus with ACE2 causes the loss of ACE2 on the cell surface, which generates an elevation of the levels of angiotensin II. Additionally, it engenders a decrease of angiotensin 1-7 levels, contributing to cell function loss, inflammation, and fibrosis, as happens in lung tissue.^2^^,^^3^ The second mechanism is associated with a cytokine storm caused by an exaggerated auxiliary type 1 and 2 T lymphocyte response, accompanied by respiratory failure and generalised hypoxia, which compromise the viability of myocardial tissue.^9^

These events should raise concern about the medium- and long-term prognoses of individuals with basic cardiovascular diseases, as well as those with cardiovascular risk factors. Due to an inflammatory process as severe as COVID-19, such subclinical conditions are expected to have progressed, and may debut with a sudden clinical picture. Likewise, scientific work should further investigate the precise pathophysiological mechanisms that trigger cardiovascular, cerebrovascular, and renal complications, since the involvement of these organs generates morbidity, mortality, and disability.

## Source of funding

This research did not receive any specific grant from funding agencies in the public, commercial, or not-for-profit sectors.

## Conflict of interest

The authors have no conflict of interest to declare.

## Ethical approval

This study does not contain any experimental research with humans or animals performed by any of the authors. Ethical approval was exempted.

## Authors' contributions

All authors equally contributed to all aspects of the research. All authors have critically reviewed and approved the final draft and are responsible for the content and similarity index of the manuscript.
